# Public Discourse Against Masks in the COVID-19 Era: Infodemiology Study of Twitter Data

**DOI:** 10.2196/26780

**Published:** 2021-04-05

**Authors:** Mohammad Al-Ramahi, Ahmed Elnoshokaty, Omar El-Gayar, Tareq Nasralah, Abdullah Wahbeh

**Affiliations:** 1 Texas A&M University-San Antonio San Antonio, TX United States; 2 Northern Michigan University Marquette, MI United States; 3 Dakota State University Madison, SD United States; 4 Supply Chain and Information Management Group D’Amore-McKim School of Business Northeastern University Boston, MA United States; 5 Slippery Rock University of Pennsylvania Slippery Rock, PA United States

**Keywords:** pandemic, coronavirus, masks, social medial, opinion analysis, COVID-19

## Abstract

**Background:**

Despite scientific evidence supporting the importance of wearing masks to curtail the spread of COVID-19, wearing masks has stirred up a significant debate particularly on social media.

**Objective:**

This study aimed to investigate the topics associated with the public discourse against wearing masks in the United States. We also studied the relationship between the anti-mask discourse on social media and the number of new COVID-19 cases.

**Methods:**

We collected a total of 51,170 English tweets between January 1, 2020, and October 27, 2020, by searching for hashtags against wearing masks. We used machine learning techniques to analyze the data collected. We investigated the relationship between the volume of tweets against mask-wearing and the daily volume of new COVID-19 cases using a Pearson correlation analysis between the two-time series.

**Results:**

The results and analysis showed that social media could help identify important insights related to wearing masks. The results of topic mining identified 10 categories or themes of user concerns dominated by (1) constitutional rights and freedom of choice; (2) conspiracy theory, population control, and big pharma; and (3) fake news, fake numbers, and fake pandemic. Altogether, these three categories represent almost 65% of the volume of tweets against wearing masks. The relationship between the volume of tweets against wearing masks and newly reported COVID-19 cases depicted a strong correlation wherein the rise in the volume of negative tweets led the rise in the number of new cases by 9 days.

**Conclusions:**

These findings demonstrated the potential of mining social media for understanding the public discourse about public health issues such as wearing masks during the COVID-19 pandemic. The results emphasized the relationship between the discourse on social media and the potential impact on real events such as changing the course of the pandemic. Policy makers are advised to proactively address public perception and work on shaping this perception through raising awareness, debunking negative sentiments, and prioritizing early policy intervention toward the most prevalent topics.

## Introduction

COVID-19 is an infection caused by the novel coronavirus SARS-CoV-2 that is known to cause severe acute respiratory syndrome [1]. As of December 26, 2020, COVID-19 had affected 192 countries around the word, with a total of 80,416,535 reported cases and 1,757,888 resulting deaths [2]. The World Health Organization, the Center for Disease Control and Prevention, and other leading public health organizations have outlined several guidelines to mitigate the COVID-19 pandemic. These guidelines have also been reported in recent scientific studies regarding the spread of COVID-19. The success of initiatives aimed at reopening the national and regional (state) economies ultimately relies on public awareness and acceptance of these guidelines for limiting the transmission of COVID-19. Among these guidelines is the importance of wearing masks.

Existing studies have shown that masks could have a substantial impact on virus transmission and wearing masks might significantly decrease the number of new COVID-19 cases [3,4]. Wearing a mask was found to be more effective than just handwashing [5]. Studies have also shown that mask-wearing diminishes disease spread by reducing the transmission probability per contact. Wearing masks in the public is most effective in stopping the spread of the virus when compliance is high [6] and presents a rational way to implement as a nonpharmaceutical intervention to fight COVID-19 [7]. Wearing a face mask can be effectively combined with social distancing to flatten the epidemic curve [7]; it is also an effective method of adequate isolation for individuals [8]. Ma et al [9] found that N95 masks, medical masks, and even homemade masks could block at least 90% of the virus in aerosols. Wang et al [10] found that the necessity of wearing masks during the COVID-19 pandemic has been underemphasized by the public. Despite its importance, as supported by scientific evidence, wearing masks has stirred up a significant debate, particularly in the United States.

With millions of people forced out of public spaces, many conversations about wearing masks take place on social media [11]. Popular social media platforms, including Twitter, have enabled new channels for users to share information and their experiences [12]. These platforms provide efficient methods of information access for health surveillance and social intelligence [13-15], and they have a growing popularity for sharing and debating scientific information [16-18]. Several studies have used Twitter as a data source to demonstrate the potential to identify the public’s reactions to a variety of public health concerns, including the opioid crisis [19], marijuana [20-22], and vaping [23]. However, there are limited studies that have examined the public discourse against wearing masks on social media and its potential relation to the rise of COVID-19 cases.

With plenty of evidence supporting the effectiveness of masks in mitigating the spread of COVID-19, the vigorous public debate about masks is still ongoing [24]. Accordingly, in this study, we aim to provide insights into factors and topics encompassing the ongoing (and sometimes contentious) debate surrounding mask-wearing. Specifically, our research objective is to investigate the topics associated with the public discourse against wearing masks. The study also analyzed trends over time for each topic, with a particular emphasis on the relative volume for each topic, and the spikes in volume. Further, we studied the relationship between the anti-mask discourse on social media and the number of new COVID-19 cases. The time-lagged cross-correlation (TLCC) is used to identify directionality between two signals—volume of tweets and COVID-19 cases—to determine which signal occurs first by analyzing cross-correlations, wherein a peak correlation may have a different offset if one signal leads another. The analysis provided insights into the potential relationship between the cyber world represented by activities on social media and the physical world represented by individuals’ actions and possibly reflected in increased infection rates. Such an understanding is needed as governments and public health officials grapple with reopening the economies, and keeping them open, in a manner that does not aggravate the COVID-19 pandemic as a public health crisis of epic proportions.

## Methods

**Figure 1:**
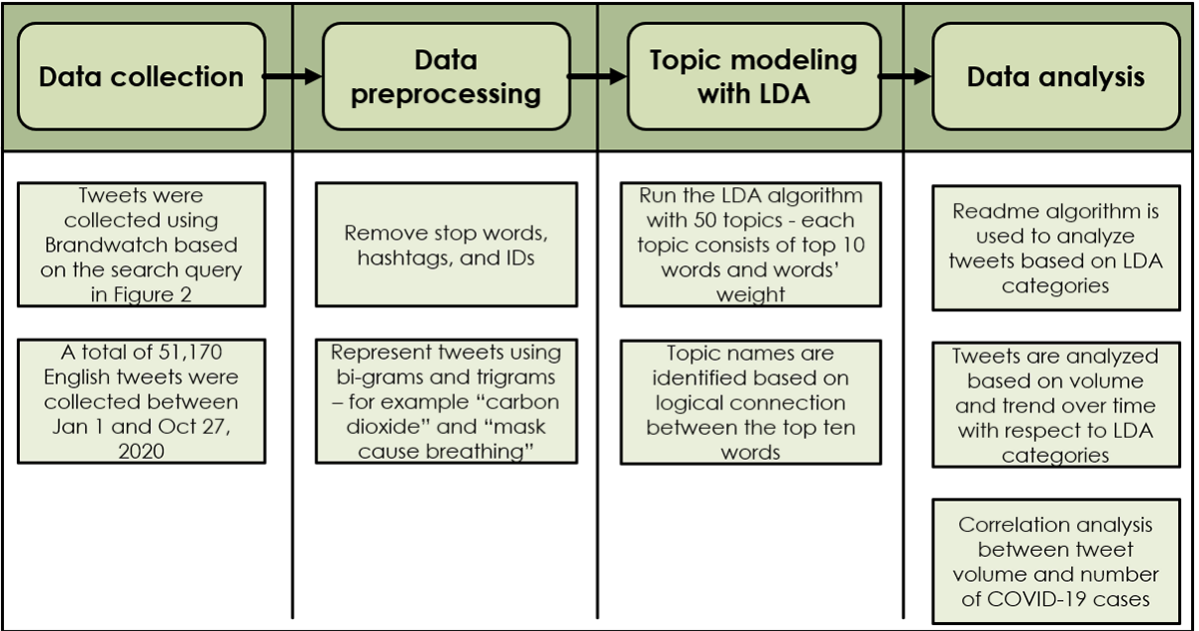
Methodology for mining social media. LDA: latent Dirichlet allocation.

Figure 1 shows the methodology adopted in this study for mining social media. The first stage involved data collection. The researchers agreed on a time period of interest to collect data and keywords (ie, hashtags) to search for relevant tweets. Second, the tweets collected were preprocessed by removing stop words, keywords with IDs, and hashtags; these were then represented using bi- and trigrams. Third, a topic modeling technique, the latent Dirichlet allocation (LDA) algorithm [25], was used to analyze the preprocessed tweets to identify the prominent topics or categories in the posts. Finally, a social media analytics tool by Brandwatch was used to analyze the frequency and track the volume of the predefined categories over time. Brandwatch employs unsupervised and supervised machine learning techniques and a text analysis model developed by Hopkins and King [26].

**Data Collection**

**Figure 2:**
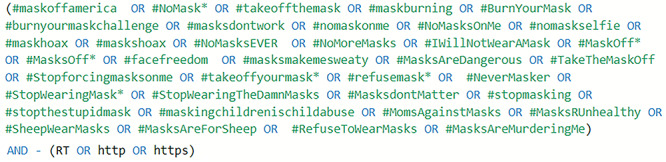
Hashtags and search query used for data collection.

Our target social media platform for data collection was the microblogging platform Twitter. Initially, we identified all hashtags against wearing masks that were being actively used on Twitter. Next, using Brandwatch with the search query shown in Figure 2, we extracted all tweets for the identified hashtags between January 1, 2020, and October 27, 2020. A total of 51,170 English tweets were collected. The hashtags were identified by reviewing the literature [27] as well as by exploring similar trending hashtags used against wearing masks on websites such as hashtags.org [28] and hashtagify.me [29]. A key advantage of using a social media analytics platform such as Brandwatch is that it provides access to the “Twitter firehose” (ie, every public tweet ever posted on Twitter in any language and from any geographic location that meets the search criteria).

For comparing the volume of tweets against wearing masks and the number of COVID-19 cases, we collected a time series of the daily number of newly reported COVID-19 cases in the United States from January to October 2020 by using data from John Hopkins University [30]. We also collected data on new COVID-19 cases reported daily in the USA from January 22 to October 27, 2020.

In acquiring data from Twitter, we considered all the common regulatory concerns that arise with social media research. Specifically, the study conforms with federal regulations on research about human subjects by using only public information that requires no interaction with the poster [31]. Moreover, the use of Brandwatch ensured that the study conformed with all the common ethical questions raised when performing web mining [32].

### Data Preprocessing

We excluded retweets and addresses to focus solely on personal opinions or statements. First, the collected tweets were preprocessed by removing stop words as well as keywords with IDs and hashtags. Second, tweets were represented using unigrams, bigrams, and trigrams, such as “results,” “lab results,” and “check test results.” Word-level n-grams features were selected to represent tweets instead of the bag-of-words (ie, single words) feature because the latter has two major drawbacks: (1) they lose the ordering of the words and (2) they ignore semantics of the words [33,34].

### Data Analysis Using the LDA Algorithm (Unsupervised Learning)

To discover the abstract “topics” that occur in the collected posts, we ran a topic mining model, specifically the LDA algorithm, with 50 topics. Given a set of documents, *D* = {*d*_1_, *d*_2_, … , *d*_n_}; a number of topics, *T* = {*t*_1_, *t*_2_, … , *t*_m_}; and a number of words in each topic, *W* = {*w*_1_, *w*_2_, … , *w*_k_}; the LDA algorithm generates the following:

- A *D*×*T* matrix with *n*×*m* size, where the weight *w*_i,j_ is the association between a document *d*_i_ and a topic *t*_j_ [35].
- A *T*×*W* matrix with*m*×*k* size, where the weight *w*_i,j_ is the association between the topic *t*_i_ and a word *w*_j_[35].

The corresponding reproductive process is shown below [35,36]:

1. For each topic *t* ∈ {1, …, *m*},
  - generate a probability distribution over words
    *βt* ~ Dirichlet (η)
2. For each document *d*,
  - generate a vector of the topic probability distribution
    θ*d* ~ Dirichlet (α)
  - For each word *w*_i_ in document *d*,
    - generate a topic assignment
      *z*_i_ ~ Multinomial (θ*d*);
    - generate a word
      *w*_i_ ~ *Multinomial* (βzi)

*βt* is the word distribution for topic *t*, and *θd* is the topic distribution for document *d*. The notations η and α are model parameters.

Topic models are statistical based models for uncovering the main themes (ie, set of topics) that depict a large and unstructured collection of documents. Topic models make it possible to summarize textual data at a scale that cannot possibly be tackled by human annotation. In this study, we chose the LDA algorithm [25] owing to its conceptual advantage over other latent topic models [35-38].

The 50 topics from the LDA were labeled by first author and validated by second author. The identified topics were further analyzed and grouped into 10 representative categories. The grouping was done based on semantic similarities between the topics identified. For example, the topics “build herd immunity,” “herd Immunity,” and “build immune system” could be grouped into in one main topic, namely, “herd immunity and dependency on the immune system.” Overall, we discovered and collected 10 different categories.

### Analysis of Tweets Using Categories Obtained (Supervised Learning)

Brandwatch employs ReadMe, a supervised algorithm developed by Hopkins and King [26]. The algorithm is particularly suited when the objective is to know the proportion of all posts that fit in specific categories. Rather than calculating these proportions based on the categorization of individual posts, ReadMe gives approximately unbiased estimates of category proportions even when the optimal classifier performs poorly [26].

The ReadMe algorithm requires the researcher to hand-code a “training set” of documents into a set of predefined categories. In this study, the tweets represent the set of documents and the predefined categories are obtained using the LDA algorithms. The authors hand-coded 20 tweets into each predefined category obtained from the LDA and then ran the ReadMe algorithm iteratively on the remaining posts, ensuring that the examples clearly outline each category. Then, based on the training phase, the algorithm builds a model that can automatically assign the remaining tweets into categories and obtain the total number of tweets in each category. Brandwatch automatically generates the trends of tweet volumes over time.

### Analyzing the Relationship Between the Tweet Volume and the Number of COVID-19 Cases

To analyze the relationship between the volume of tweets against mask-wearing and the daily volume of new COVID-19 cases, we plotted two time-series over the time span from January to October 2020 and calculated the Pearson correlation coefficient, which measures how two continuous waves co-vary over time and indicate the linear relationship as a number ranging from –1 (negatively correlated) to 0 (not correlated) to 1 (perfectly correlated) [39]. The correlation is a snapshot measure of global synchrony. Although the Pearson correlation coefficient provides a very simple way to compute both global and local synchrony, it does not provide insights into signal dynamics such as which signal occurs first or which can be measured via cross-correlations. A TLCC can identify directionality between two signals such as a leader-follower relationship. We can get a sense of which signal occurs first by looking at cross-correlations. A TLCC is measured by incrementally shifting one time-series vector and repeatedly calculating the correlation between two signals. If the peak correlation is at the center (offset=0), this indicates that the two time-series are perfectly synchronized at that time. However, the peak correlation may have a different offset if one signal leads another [40]. To analyze the relationship between the two time-series, the volume of tweets against mask-wearing, and the daily volume of new COVID-19 cases, we calculated the Pearson correlation coefficient and TLCC in Python using the SciPy package.

## Results

### Tweet Distribution and Categories

#### Overview

**Figure 3:**
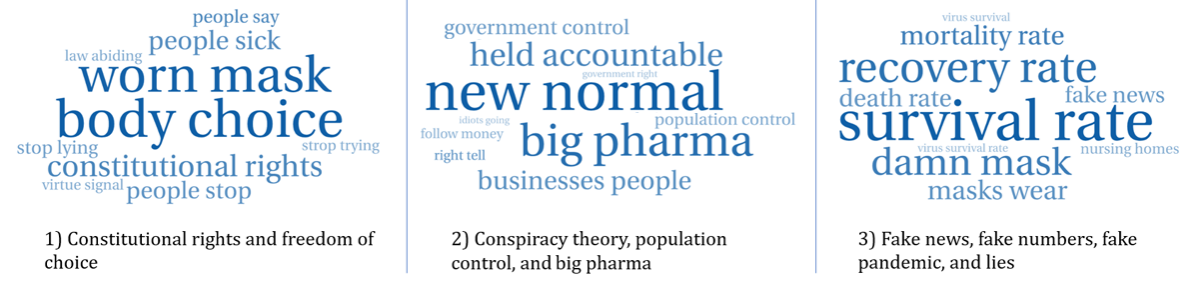
Word cloud for the most common categories identified: (1) constitutional rights and freedom of choice; (2) conspiracy theory, population control and big pharma; AND (3) fake news, fake numbers, fake pandemic, and lies.

**Figure 4:**
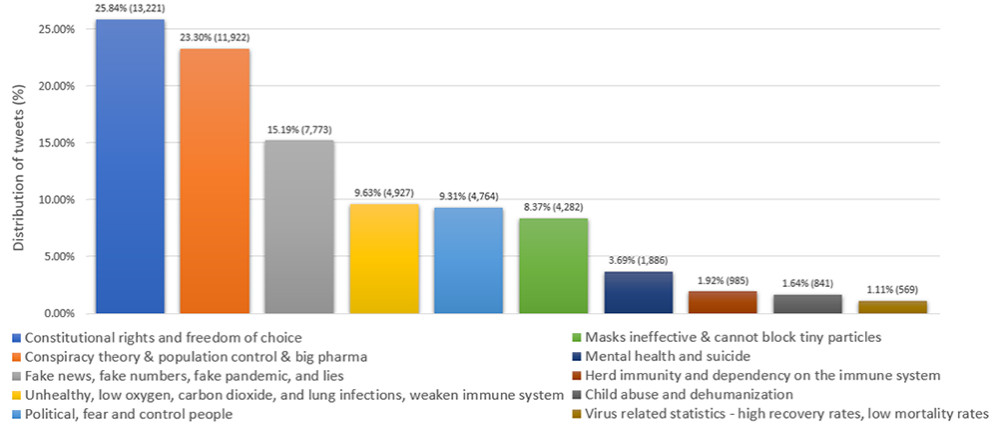
Distribution of 51,170 tweets across the top 10 categories obtained using the latent Dirichlet allocation model.

A total of 51,170 tweets were analyzed with respect to categories identified from the LDA model. These categories were mainly related to (ordered per their frequency in posts) (1) constitutional rights and freedom of choice; (2) conspiracy theory, population control, and big pharma; (3) fake news, fake numbers, fake pandemic, and lies; (4) unhealthy, low oxygen, carbon dioxide, lung infections, and weakened immune system; (5) political, fear, and control people; (6) masks ineffective and cannot block tiny particles; (7) mental health and suicide; (8) herd immunity and dependency on the immune system; (9) child abuse and dehumanization; and (10) virus-related statistics (high recovery rates and low mortality rates). Figure 3 shows the word clouds for the first three categories. The distribution of the tweets over the categories identified is shown in Figure 4.

**Figure 5:**
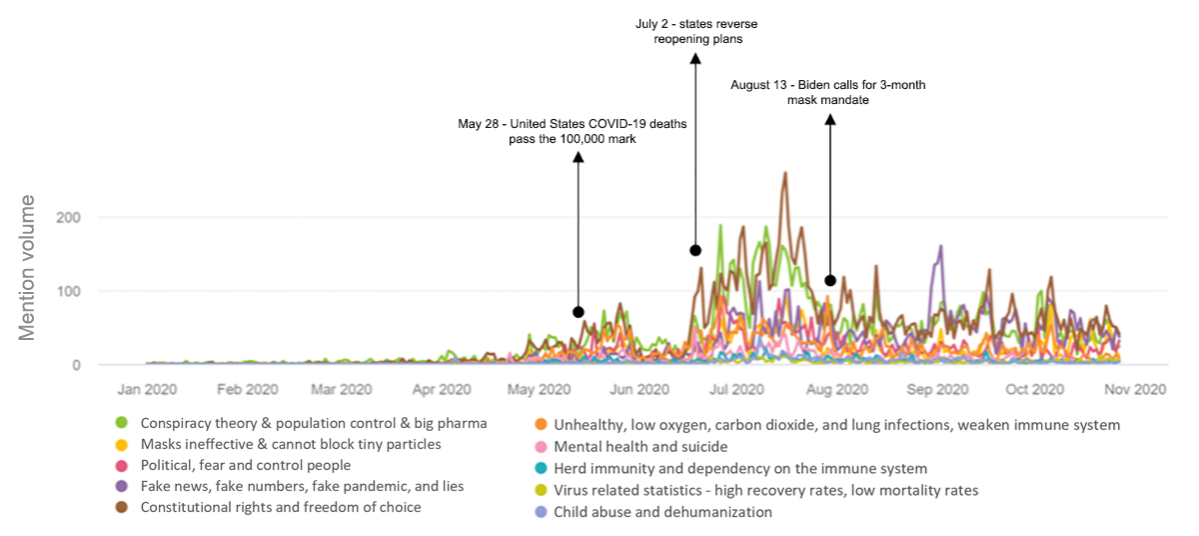
Volume of tweets and trend analysis over 10 categories based on the latent Dirichlet allocation model as well as three significant milestones during the pandemic between January 1, 2020 and October 27, 2020.

Figure 5 shows the volume of tweets over time by category. Overall, the number of tweets posted increased with time, with the highest volume of tweets recorded in July 2020. Between April 8, 2020, and May 29, 2020, a total of 15 states issued a mask mandate, which could be related to the spike in tweets posted on masks between April and the beginning of July 2020. Furthermore, between June 18 and August 11, 2020, another 20 states issued mask mandates [41]; this could explain the increase in tweets posted about masks between late-June and mid-August. Figure 5 also shows three relevant milestones between May and August 2020 [42]. These three milestones are related to the number of deaths reported in late-May, states reversing reopening plans, and the call for 3-month mask mandates. These milestones could also relate to the increasing number of Twitter posts on masks. Furthermore, after August 13, 2020, we noted consistent debates on masks across all post categories.

Figure 5 also shows that more tweets were posted as governments and public health officials relaxed the lockdown restrictions but requested people to continue wearing masks. The number of tweets posted about constitutional rights and freedom of choice increased noticeably, followed by tweets about conspiracy theory, population control, and big pharma. The following paragraphs provide a synopsis of each of the categories of tweets posted.

#### Constitutional Rights and Freedom of Choice

Our results revealed several reasons why some Americans refuse to wear face masks despite the overwhelming evidence that wearing masks saves lives. One important reason discussed during the study period was constitutional rights and freedom of choice. Many say mandatory masks violate their constitutional right and freedom of choice. An example tweet is shown below:

Dear #****, I am an American citizen with constitutional rights. I have the right & freedom to choose #NoMask. If u try to enforce this ridiculous order, I will sue your ass 2 hell & back. Kentucky is a #redstate & you don't belong. GTFO. Signed a pissed of Kentucky girl

#### Conspiracy Theory, Population Control, and Big Pharma

Americans also discussed concerns related to conspiracy theory, population control, and big pharma. They believed that COVID-19 was human-engineered. Example tweets are shown below:

Won't have to listen to people blabbering on about their latest favourite conspiracy theory

You can have a ridiculous opinion. Democrats follow blindly, I do not. **** IS Big Pharma. Masks = Control = Submission that will lead to mandatory inoculation of a genetically modifying vaccine. If dems win, we all lose. #MasksOffAmerica

#### Fake News, Fake Numbers, Fake Pandemic, and Lies

Many also believed the pandemic is fake and there was fake news, misinformation, and lies spread about COVID-19. Example tweets are shown below:

@**** Seasonal flu kills more people EVERY year. You and the fake news media are losing credibility FAST. #nomasks #nonewnormal

@**** So how many other false positives are out there...this makes the numbers even more questionable

#### Unhealthy, Low Oxygen, Carbon Dioxide, Lung Infections, and Weakened Immune System

Tweets posted also discussed the health impact of wearing masks. Many believed masks limit oxygen intake and cause rebreathing of carbon dioxide, which can lead to lung disease and weaken the immune system. Example tweets are shown below:

Wearing it blocks oxygen and recycles carbon dioxide and carries the bacteria to your respiratory system. #nomasks

Masks weaken the immune system. Masks allow oral bacteria to affect gums, throat & lungs. Masks limit oxygen intake. Masks cause rebreathing of carbon dioxide

#COVID-19 #NoMasks Hypercapnia is generally caused by hypoventilation, lung disease, or diminished consciousness

#### Political, Fear, and Control People

Another topic discussed by Americans on Twitter was fearmongering. Many users believed that politicians and media have only focused on the numbers that present a negative picture of the COVID-19 pandemic rather than a more balanced and honest overview of the case numbers. Example tweets are shown below:

@**** Nor do they speak about the low death rate. They want us living in fear. Fear controls the masses! #SheepNoMore #MaskOff

FEAR MONGERING!!! THIS IS WHAT IT LEADS TO! ENOUGH! NO MORE MASKS!!

#### Masks Ineffective and Cannot Block Tiny Particles

Many users also had an opinion that masks are ineffective and cannot block tiny particles. Example tweets are shown below:

People wearing #masks and shaming others for NOT wearing them though all #science deems them almost totally ineffective in protecting against the nano particles of the coronavirus. #DumbPandemicDecisions #Masks4All #MasksOff

@**** says masks are ineffective to stop the virus. Why is there a state execution/executive order now to mandate masks? #NoMasks #ControlRemedy

#### Mental Health and Suicide

Many users thought that wearing mask could also have impact on the mental health of people and could lead to suicidal thoughts. Example tweets are shown below:

they are causing a severe mental health issue. #NoMasks #MasksOff

Masks are causing horrible harm with the mental health of children. Stop wearing them before these damages are irreversible! #NoMasks #MasksOffArizona

Masks are causing serious mental health issues in children. Stop with the masks before it’s too late! #MasksOff

Where is the **** physician saying that this lockdown needs to end b/c suicide is up? Mental health has been ignored completely

#### Herd Immunity and Dependency on the Immune System

People should not be forced to wear masks in order to build herd immunity and maintain a healthy and strong immune system. Example tweets are shown below:

It's time we focus on REAL solutions like herd immunity. #NOMASK for me. @****

You need INTERACTION with people and #NoMasks to maintain a healthy immune system #OpenAmericaNOW #OPenHawaiiNow

I will NOT wear a damn mask!! It is my right to come in to contact with germs that strengthen my immune system!

#### Child Abuse and Dehumanization

Asking children to wear masks was considered child abuse according to many Twitter users in USA. Example tweets are shown below:

Masking children is child abuse! Kids are not at risk and not carriers of the virus! Kids need to see and communicate clearly. They need to see facial expressions. A mask desensitizes kids! #maskingchildrenischildabuse

Mandating our young children to wear a mask for 7hrs per day while attending school is tantamount to child abuse. #OpenTheSchools #NoMasks

Masks in this case are a tool for soft torture and dehumanization #NoMasks

#### Virus-Related Statistics (High Recovery Rates and Low Mortality Rates)

Twitter users also discussed that the high recovery and low mortality rate of the virus that make wearing mask not necessary. Example tweets are shown below:

I will not comply and wear a useless mask that has potential health risks to me for a virus that has a 98% recovery rate. #NoMask

COVID-19 Mortality Rate in CA is .00006925% that means 99.999932% are forced 2 destroy R lives 4the weakest virus on the planet! Stop Quarentining the Healthy, Open up Businesses & only Quarantine the Sick! #UnMaskAmerica

### Tweets Versus New COVID-19 Cases

**Figure 6:**
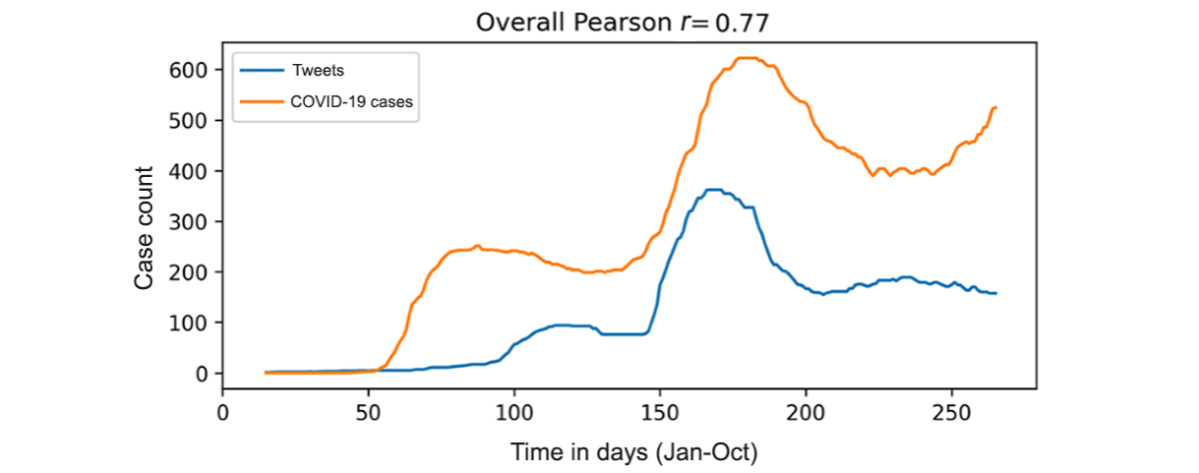
Pearson correlation of tweets against wearing masks and newly confirmed COVID-19 cases over time (days) between January 2020 and October 2020.

**Figure 7:**
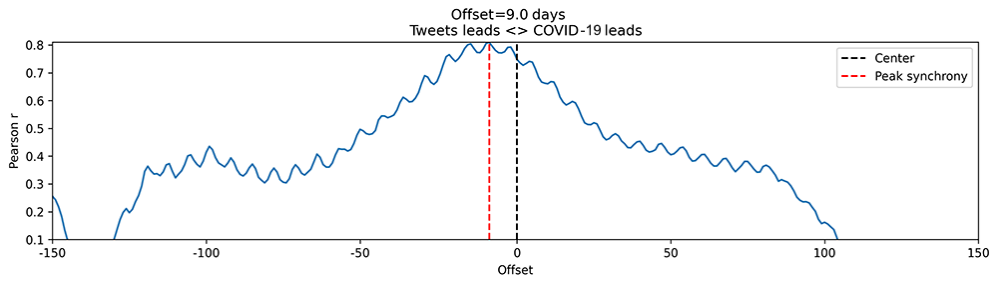
Graph illustrating the 9-day lead in the volume of tweets against mask-wearing compared with new COVID-19 cases by 9 days (study period: January to October 2020).

Figure 6 depicts the volume of tweets against wearing masks and the number of newly reported COVID-19 cases over the study period. The two time-series exhibit a high positive Pearson correlation (*r*=0.77). Since information about directionality between the two waves—leading and following—cannot be interpreted solely from this data, we further studied the relationship between both waves (Figure 7). Overall, the results show a 9-day lead for tweet volume over the number of new COVID-19 cases. This 9-day lag is considered comparable to the number of days after which people can develop COVID-19 symptoms. According to a previous study, approximately, 97% of people infected with COVID-19 developed symptoms within 12 days after exposure [43].

## Discussion

### Principal Findings

This study analyzed the negative stance regarding masks on social media, the specific themes within this discourse, and how this discourse could be associated with the prevalence of new COVID-19 cases. The study reported Twitter users’ concerns related to constitutional rights and freedom of choice, conspiracy theory, misinformation, health issues, fearmongering, and other concerns related to the use of face masks during the COVID-19 pandemic. Furthermore, the time-series analysis demonstrated a strong correlation between the number of tweets posted against mask-wearing and the actual number of COVID-19 new cases, with the volume of negative tweets leading the number of newly reported COVID-19 cases by 9 days.

The study findings emphasize the potential relationship between social media behavior and its manifestation in the physical world. Such findings highlight the importance of listening to social media and proactively reacting to public perception in fighting COVID-19. Lyu and Wehby [44] showed that mask mandates in a number of states were associated with lowering infection rates by 0.9%-2% after wearing masks for 1-21 days. However, when the government mandates mask-wearing in public, many people feel their constitutional rights and freedom of choice are being violated [45]. As a result, there is a need to increase awareness about the fact that wearing masks can protect others from contracting COVID-19 even though they do not fully protect the person wearing the mask from the infection [46]. The government should also address the challenges faced by implementing a balanced mask-wearing mandate that considers protecting people's lives while also protecting their freedom of choice [47].

Social media platforms have been used to spread fake news, lies, and conspiracy theories, all of which have a strong impact on people and society [48]. As a result of such an impact, the public is less likely view actions like wearing masks as a necessity to mitigate the spread of the virus during a pandemic [49]. Therefore, it is crucial that, as we seek to control the spread of COVID-19 and future viruses, we develop policies to fight against misleading and damaging conspiracy rhetoric. Similarly, there should be policies in place to combat fake news, lies, and misinformation, especially on social media, which could negatively affect the public’s trust in science [49].

Health care professionals should actively engage in the conversation with the public in order to discuss scientific evidence supporting the importance of wearing a mask and debunk rumors on social media that promotes discussions related to masks causing low oxygen levels or lung infections. They should also discuss evidence and guidelines such as “wearing a mask does not raise the carbon dioxide (CO_2_) level in the air you breathe” [50] and “people aged 2 and older should wear masks in public settings and when around people who don’t live in their household” [50] to increase awareness regarding the effectiveness of masks in protecting the wearer from inhaling and spreading airborne particles.

Children of specific age groups should be encouraged to wear masks to protect them from COVID-19. However, protecting these age groups only by using a mask could prove very difficult [51]. To overcome these challenges, there is a need to advocate for parental involvement and support for the initiatives aimed at increasing mask-wearing among children [51]. Children should be encouraged to “take off their masks to breathe in fresh air after wearing masks for a certain amount of time,” and they should not wear masks in certain cases, such as while exercising [51]. In the case of noncompliance, it would be a better option for children to not wear masks and follow other measures to reduce infection risk and remain at home [51].

Following an empathetic approach to motivate people to wear masks and adhere to physical distancing could be an effective alternative [52] for fearmongering that focuses only on presenting a negative picture of the COVID-19 pandemic [53]. In addition, policy makers could use positive messaging to curb the spread of fear while still maintaining a transparent and accurate depiction of the situation [53].

With physical, mental, social, and economic burdens imposed by the pandemic, many populations may experience increased suicide risk [54]. Furthermore, the prevalence of anxiety, depression, posttraumatic stress disorder, and stress was reported to have increased in a number of countries during the COVID-19 pandemic [55]. Data analysis and event surveillance conducted during the first 6 months of the pandemic have shown impacts on suicide risk [54]. As a result, knowing the facts about masks and containing the spread of rumors can reduce stress and the adverse impacts on people’s mental health [56]. Finally, since many people believe that herd immunity is the best solution to this public health crisis and to strengthen their immune systems, a scientific and fact-driven view should be shared with the public explaining why herd immunity is not an ideal solution as has been reported by many researchers [57].

By carefully analyzing social media posts, policy and decision makers are in a better position to tailor public health awareness campaigns to respond to specific themes and thereby improve their effectiveness in a crisis situation such as the COVID-19 pandemic. Thus, exploring the categories of tweets surrounding the topic of mask-wearing during the COVID-19 pandemic may help reveal a number of insights that could help better design and implement awareness campaigns.

### Limitations and Future Work

This study has some limitations that could be addressed in future research. First, although we identified a very strong correlation between the increase in the volume of tweets against wearing masks and the rise in the number of COVID-19 cases, we cannot claim causality, as the rise in COVID-19 cases could be attributed to population density, government-enforced lockdown restrictions, and other factors that are beyond the scope of this study. Second, the study focused on analyzing English tweets in the United States. Future studies need to address and compare the public discourse on masks across different social media platforms and in different countries. Third, given the number of tweets collected and the focus on Twitter as a data source, the public discourse might not reflect the actual public opinion against masks. According to Wojcik and Hughes [58], Twitter has been found to have much younger audiences, with the most prolific 10% of users creating 80% of all tweets published. Finally, we did not separately analyze the opinions of Twitter users against masks in the early and later stages of the pandemic. Such analysis could unmask other important trends that are not discussed in this paper.

### Conclusions

In this study, we analyzed tweets against wearing masks on social media to understand topics, insights, and information about user-reported issues. We used data analytics to identify trending themes and topics of concern by the public about wearing face masks. The most discussed issues were related to the constitutional rights and the freedom of choice, conspiracy theory, misinformation, health issues, fearmongering, and the ineffectiveness of masks, followed by issues related to mental health, herd immunity, child abuse, and virus-related statistics. Another key finding of this study is that it highlights the strong correlation between the increase in the volume of tweets against wearing masks and new COVID-19 cases and the lead of negative tweets published in comparison with the rise in new COVID-19 cases in the time-series analysis. In effect, these findings demonstrated the impact of social media not only on people’s opinion or perceptions about public topics but also the potential impact on real events such as changing the course of the pandemic. The significance and implication of this research transcends the COVID-19 pandemic, as it demonstrates the importance of social media mining and its potential to support public health–related policies and decisions. Government officials and decision makers could tailor and fine-tune public awareness campaigns and prioritize policy interventions toward the most discussed topics. In case of a future massive-scale health crisis such as the COVID-19 pandemic, government officials and policy makers could leverage social media analytics and surveillance as important tools in proactively responding to the impending crisis. Policy makers need to proactively address public perception and work on shaping this perception through raising awareness, debunking negative sentiments, and adopting early policy intervention to steer the wheel towards public acceptance of more precautionary measures and thereby containing the situation.

## Abbreviations

LDA: latent Dirichlet allocation
TLCC: time-lagged cross-correlation
